# Psychological and social determinants of adaptation: the impact of finances, loneliness, information access and chronic stress on resilience activation

**DOI:** 10.3389/fpsyg.2024.1245765

**Published:** 2024-02-26

**Authors:** Leia Y. Saltzman, Tonya Cross Hansel

**Affiliations:** School of Social Work, Tulane University, New Orleans, LA, United States

**Keywords:** resilience, mental health, loneliness, chronic stress, finances

## Abstract

**Background:**

Many people who face adversity, such as disasters, demonstrate resilience. However, less is known about reactions to large scale disasters with longer recovery periods. The concern is that protracted disasters may result in more chronic or accumulated stressors with an uncertain or unknown end point and can exhaust the natural coping methods and ability to rebound. Thus, understanding patterns of longer-term disaster recovery, inclusive of resilience, is needed. Further resilience is not individual specific rather social determinants, such as support networks and available resources, are contributing factors.

**Methods:**

The purpose of this study is to improve understanding of mental health and resilience during increased stress, we aim to identify profiles of adaptation and psychological and social determinants that predict membership within predominant symptom groupings. We conducted an exploratory cross-section study (*N* = 334) with two phases of multivariate analysis. Latent profile models were estimated to identify groups based on depression, anxiety, and resilience scores. The second phase included a step-wise multinomial logistic regression to predict class membership.

**Results:**

We identified four distinct groups: 33% of participants were categorized as anxious, 18% depressed, 9% comorbid, and 40% with above average levels of resilience. Psychosocial factors such as demographics, trauma history, information access, loneliness, and lack of financial resources predicted poorer mental health outcomes and lower resilience.

**Conclusion:**

This study identified factors that contribute to overall wellbeing despite chronic stressors. Social determinants of adaptation, found in this study population, include loneliness, finances, and information access. The findings from this study support the need for both psychological and social adaption supports, inclusive of mental health treatment, to strengthen resilience activation.

## Introduction

For decades we have understood that human systems have the capacity to regulate stress and that weathering stressors is a healthy biological and psychological process. However, when stressors compound and move to a more chronic state of stress reactions, physical and mental health response systems can become overwhelmed, known as one’s allostatic load (Felitti, 2002; McEwen, 2004). Yet the protective ability of chronic stress for acute stress was less understood, as well as the situational constructs that facilitate or hinder defense mechanisms (McGonagle and Kessler, 1990). Over the years, we have synthesized the literature around this ordinary magic (Masten, 2001) as adaptive capacity or psychological resilience; however, exploration around the determinants of the resilient process are still needed (Southwick et al., 2014).

### Psychological resilience

Research on disasters have progressed the science around psychological resilience, as they are specific measurable moments in time. Studies show that the majority of persons who experience adversity demonstrate the ability to *bounce back* from the trauma and disruption such as disasters (Hansel et al., 2020). The ability to rebound eventually leads to acceptance and incorporation of the emotional response into their new normal—re-envisioning or re-establishing a vision for the future (Norris et al., 2008). However, large scale disasters with longer recovery periods, and thus more chronic or accumulation of stressors for an uncertain or unknown end point can exhaust the natural coping methods and the ability to rebound. Previous trauma, bereavement, and loss (Osofsky et al., 2011) and financial setbacks (Drydakis, 2015) are all risk factors that should be of concern in the context of prolonged sequela. As such, increased anxiety and depression are common following disasters (Norris et al., 2002; Kessler et al., 2006) but this does not mean the absence of resilience. For example, studies following the Gulf oil spill in 2010 found increased mental health problems met with high resilience scores (Osofsky et al., 2011; Shenesey and Langhinrichsen-Rohling, 2015). Thus, understanding patterns of recovery, inclusive of resilience, are needed. It is likely that survivors will experience resilience, however the patterns of recovery, including resilience, is still not well understood – particularly when considered over time.

### Social resilience

Social support is a strong predictor of resilience following disaster and trauma (Hall et al., 2010; Xu & Ou, 2014; Saltzman et al., 2017; Matthews et al., 2019; Osofsky et al., 2022); likewise, disruption of many support systems can challenge resilience (Cattan et al., 2005; Xu et al., 2011; Valtorta et al., 2016). In a systematic review of general social isolation, Leigh-Hunt et al. (2017) found associations with both poorer mental and physical health, yet the impact on resilience is not as well understood. Similar to social support, interdependence, or the belief that one’s actions are part of collective community change, has been noted as an important protective measure (Bollyky and Bown, 2020; Lo & Hsieh, 2020). Another study (Vashdi et al., 2020) noted the buffering effect of interdependence on more negative outcomes and the importance of connectedness as a mechanism toward collective resilience.

### Resilience activation framework

Resilience was first used to describe adaptations of the ecosystem and overtime extended to human systems and the built environment (Alexander, 2013). While scholars have tended to view them as siloed, research is beginning to demonstrate the connectedness of both psychological and social resilience. Abramson et al. (2015) provide a conceptual framework for resilience activation that considers human, social, economic and political capital at both the community and individual levels. Thus, resilience is not individual specific outcome but rather social determinants, such as support networks and available resources, are contributing attributes. A framework for resilience activation allows for complex and systematic factors that contribute to resilience, yet research is needed to support this theoretical model.

Similarly, collective resilience has been defined as a set of adaptive capacities that allow for consideration of and emphasize the importance of both individual and social attributes in the activation of resilience (Norris et al., 2008; Pfefferbaum, et al., 2008; Picou et al., 2009; Renschler et al., 2010). A collective framework also has the benefit of addressing some of the controversies around resilience regarding how it is defined and who is deemed resilient (Masten, 2014). Specific to disaster, the idea of bouncing back to unjust systems are also worth noting and require more consideration through a collective resilience framework. Clearly more research is needed to understand the psychological and social determinants that contribute to the resilience activation framework (Abramson et al., 2015).

The current study combines the frameworks of resilience activation and social resilience to explore the multi-system factors that influence psychological profiles of adaptation (including resilience) following a protracted disaster. In doing so, the current study aims to underscore the interaction of individual characteristics and contextual, social, and environmental factors that influence trajectories of adaptation. This work adds to the literature supporting both resilience activation and social resilience frameworks and expands the body of literature to include the application to large scale protracted disasters including public health crises.

## Purpose

Supporting early studies of chronic stress, recent scholars have noted that long-term resilience may be a common outcome following disasters (Buheji, 2020; PeConga et al., 2020) and may be particularly true for individuals who have weathered prior traumatic experiences (Hansel et al., 2015; Blanc et al., 2020). The long-term nature of the COVID-19 pandemic and subsequent stressors, isolation, and social disruption (Lau et al., 2005; Classen and Dunn, 2012; Hempstead & Phillips, 2015; Saltzman et al., 2020), present the opportunity to study the resilience activation framework. Unique to biological disasters and similar to technological disasters, there is no clear end point. However, the United States ending the declaration of the public health emergency on May 11, 2023 (Center for Disease Control and Prevention, 2023) suggests the recovery phase is looming. Services to support coping and improve wellbeing are critical to recovery, yet the type of service is dependent on symptom profiles and accompanying levels of resilience.

The purpose of this study is to explore psychological and social determinants of adaptation, to identify contributors to resilience activation. We aim to improve understanding of mental health and resilience during increased stress, by identifying profiles of adaptation and factors such as, personal and social determinants, that predict membership within predominant symptom groupings. This approach aligns with previous research that utilizing latent structures such as confirmatory factor analysis (Diotaiuti et al., 2021b) and structural equation modeling (Diotaiuti et al., 2021a) to explore complex psychosocial structures such as resilience, even within disaster contexts (Diotaiuti et al., 2021b). Consistent with other studies (Osofsky et al., 2022), we hypothesized a 4-factor (depressed, anxious, comorbid, and resilient) solution and further explore predictors of group membership. Through identification and prediction of mental health subgroups, services and outreach can be targeted toward the needs of each group to strengthen resilience activation (Osofsky et al., 2017; Cui et al., 2018).

## Methods

The study began in the United States on April 7, 2020 and continued through December 16, 2020. Study participants were recruited through Tulane University School of Social Work’s website and media promotions and completed through a Qualtrics link. Participants viewed a welcome letter and gave virtual consent by continuing the survey. Being an adult (18 years of age or older) and having access to the technological platform were the only limiting factors. We informed participants they could skip or stop at any time—there was no compensation. Ethical approval for the study was provided by Tulane University’s Human Research Protection Office.

### Measures

Descriptive statistics for study measures and variables are presented in Table 1. Resilience was measured using the 2-item Connor Davidson Resilience Scale (Vaishnavi et al., 2007), asking participants if they (1) are able to adapt to change and (2) tend to bounce back from setbacks (*α* = 0.73, *M* = 8.23, SD = 1.37). General Anxiety Disorder assesses anxiety as (1) feeling nervous, anxious, or on edge and (2) being able to stop or control worrying (Sapra et al., 2020; *α* = 0.88, *M* = 4.80, SD = 1.96) in the past 30 days. The Patient Health Questionnaire assesses depression as being bothered by (1) little interest or pleasure in doing things and (2) feeling down, depressed, or hopeless (Kroenke et al., 2003; *α* = 0.86, *M* = 3.89, SD = 1.71).

**Table 1:** Descriptive statistics on study variables.

		Min	Max	M	SD
1	Interdependence	8.0	20.0	17.1	2.4
2	Disruption	5.0	15.0	11.5	2.6
3	Stressors	0.0	10.0	3.8	1.7
4	Anxiety	2.0	8.0	4.8	2.0
5	Depression	2.0	8.0	3.9	1.7
6	Resilience	4.0	10.0	8.2	1.4
7	Viral Rate	1187.1	31119.8	12251.5	5150.7
8	Financial security	1	5	4.36	1.029
9	Community closeness	1	5	3.06	1.197
10	Food insecurity	1	5	1.40	0.904
11	Information access	1	5	4.33	0.850

### Psychosocial

Pandemic related stressors included asking if participants had experienced: loss of usual way of life, social isolation, work from home, community health concerns, children and adolescents being out of school, loss of income, personal health effects, participated in response or emergency services, loss of tourism, COVID-19 symptoms or diagnosis, loss of job or business. A stress index was created where 1 point was given for each of the experiences over the past year noted above consistent with previous research (Hansel et al., 2022). Life disruption was adapted from the Sheena Disability Scales (Sheehan et al., 1996) and included items that assessed to what degree the pandemic disrupted participants’ work or school life, social and leisure activities and family or home responsibilities activities (*α* = 0.61). Participants were also asked if they experienced a previous traumatic event (yes or no).

Social determinants included four life security questions (U.S. Department of Agriculture, 2012), including, in the last 2 weeks do participants: (1) have enough money to meet needs; (2) feel close to members of community; (3) worry whether food will run out before they get money to buy more; and (4) available information needed in day-to-day life. Interdependence was adapted for biological disaster relevance (Gerpott et al., 2018). Participants were asked to provide their level of agreement on the following indicators specific to the pandemic: (1) the outcome does not affect future interactions; (2) current behavior will have consequences for future outcomes; (3) personal behavior affects how others will behave in future situations; (4) what we do for [our actions] will not affect others; and (5) we need each other to get our best outcome. Items 1 and 4 were reverse coded for scale computation (*α* = 0.64).

### Community

Community impact was assessed by county level viral rate of COVID death and diagnosis through October 2020. Using the COVID Data Tracker, we matched viral rates to participants zip code (Centers for Disease Control, 2020). Geographic information (percentage rural) was assessed at the county level and matched to participants zip code (Robert Wood Johnson Foundation, 2018).

### Participants

The mean age of participants was 46.3 years (SD = 14.7, 19–79 years of age); 84% identified as female, 16% as male and 1% as non-binary. The majority of participants identified as white (89%), 8% identified as black or African American, 5% as Latinx, 3% as Asian, and 1% as Native American (participants could select all that apply). Median income was $60,000 -$69,999 (2019 prompted as reference). Twenty percent have a high school education, 70% had a 4-year or professional degree, and 10% had a doctorate. Over half of the participants (63%) were married or cohabitating, 25% were single, 10% divorced or separated and 2% widowed. Participants (*n* = 334) resided in Louisiana (54%), Texas (6%), California (5%), Florida (4%), Georgia (3%), Illinois (3%). Two percent or less were from each of the following states: 2% from Mississippi, North Carolina, Pennsylvania, and Virginia; 1% from Arizona, Connecticut, Iowa, Kentucky, Minnesota, Massachusetts, New Jersey, New York, Maryland, Michigan, Missouri, Ohio, Oregon, South Carolina, and Washington; and 0.3% from Alabama, Alaska, Colorado, Delaware, Indiana, Nebraska, New Mexico, Rhode Island, Tennessee, Wisconsin, and Wyoming. Participants lived in urban and rural communities, with the minimum percent living in rural communities (*M* = 9%, SD = 14%, Mdn = 3.3%).

### Statistical analysis

There were two phases of multivariate analysis; in one phase a series of latent profile models were estimated to identify unobserved groups in the data. A classify analyze approach using maximum posterior probabilities was used to assign respondents to their most likely latent class (Nagin, 2005). A new variable representing the latent class *adaptation* was then generated and used in phase two. The following measures were used as indicators of latent class membership: depression (PHQ-2); anxiety (GAD-2); and resilience (Connor & Davidson); higher values represented higher symptomology/resilience. The second phase included a step-wise multinomial logistic regression to predict class membership using a four group nominal variable representing class membership. Step one included the following intrapersonal predictors, age, marital status, race, education level, respondent sex, and income. Step two included community level data (viral rate and an indicator rural or urban residential area). Step three included the following psychosocial variables, perception of interdependence, disruption, stressors, trauma, and life security (financial, social support, food insecurity, and information access).

## Results

Goodness of fit statistics for all LPA models are presented in Table 2. Interpretability and comparative model fit indices were used to select the final four class solution model. The Akaike’s information criterion (AIC) and Adjusted Bayesian Information Criterion (BIC) both decreased from the three class solution. Entropy, that is the certainty of classification, increased from 0.81 to 0.90 between the three and four class model. Finally both the Lo–Mendell Rubin likelihood ratio test and bootstrapped likelihood ratio test were significant (ll = 74.19, *p* < 0.001; ll = −1781.93, *p* < 0.001 respectively) indicating that the four class solution improved over the model in which *k* = k-1.

**Table 2:** Goodness of fit statistics for all LPA models.

Classes	Loglikelihood (df)	AIC	Adjust BIC	Entropy	LMRT	BLRT
2	−1829.96 (10)	3679.93	3686.32	0.791	190.68^***^	−1929.41^****^
3	−1781.83 (14)	3591.66	3600.61	0.812	92.30^***^	−1829.96^***^
4	−1743.15 (18)	3522.29	3533.80	0.901	74.18^***^	−1781.83^***^
5	−1745.32 (22)	3534.63	3548.69	0.830	−4.159	-

*** *p* < 0.001.

AIC Akaike’s information criterion, Adjust BIC Adjusted Bayesian Information Criterion, LMRT Lo–Mendell Rubin adjusted likelihood ratio test, BLRT, Bootstrap likelihood ratio test.

Lower AIC and BIC indicated better fit. Higher Entropy indicates greater accuracy in allocating members to latent classes. Significant LMRT and/or BLRT indicates improvement over model in which *k* = k-1. All bootstrapped models were estimated with five draws. Five class model, BLRT was not estimated as the loglikelihood value for the model with one less class was larger than the loglikelihood value for the estimated mode.

The four classes (see Table 3) are characterized as follows: (1) predominantly depressed (*n* = 62, 18.56%); (2) predominantly anxious (*n* = 110, 32.93%); (3) comorbid (*n* = 30, 8.98%); and (4) resilient (*n* = 132, 39.52%). The predominantly depressed group was characterized by endorsing higher than average levels of depression (*M* = 5.54, *p* < 0.001), slightly above average levels of anxiety (*M* = 5.97, *p* < 0.001), and lower than average levels of resilience (*M* = 7.55, *p* < 0.001). In contrast, the predominantly anxious group was more likely endorse higher than average levels of anxiety (*M* = 5.24, *p* < 0.001), about average levels of depression (*M* = 3.87, *p* < 0.001), and slightly below average levels of resilience (*M* = 8.19, *p* < 0.001). The group most likely to endorse the highest degree of symptomology was the comorbid class where respondents endorsed above average levels of both anxiety and depression (*M* = 7.39, *p* < 0.001; and *M* = 7.67, *p* < 0.001 respectively) and below average levels of resilience (*M* = 7.60, *p* < 0.001). The final class was characterized as resilient as members of this group were more likely to report below average levels of anxiety, depression (*M* = 3.25, *p* < 0.001; and *M* = 2.28, *p* < 0.001 respectively) and slightly above average levels of resilience (*M* = 8.71, *p* < 0.001). Table 3 presents the class membership and beta coefficients.

**Table 3:** Estimates four class solution.

	Class 1 (*n* = 62)	Class 2 (*n* = 110)	Class 3 (*n* = 30)	Class 4 (*n* = 132)
Indicator	Estimates (SE)	Estimates (SE)	Estimates (SE)	Estimates (SE)
Anxiety	5.97 (0.25)^***^	5.24 (0.16)^***^	7.39 (0.18)^***^	3.25 (0.13)^***^
Depression	5.54 (0.09)^***^	3.87 (0.07)^***^	7.67 (0.09)^***^	2.28 (0.02)^***^
Resilience	7.55 (0.22)^***^	8.19 (0.12)^***^	7.60 (0.26)^***^	8.71 (0.12)^***^

Sample mean anxiety = 4.80, depression = 3.89, resilience = 8.23. *** *p* < 0.001.

### Multinominal regression models

#### Predominantly depressed group

Age was significant for the predominantly depressed classes, such that, an additional year of age decreased the relative risk of belonging to the predominantly depressed group, rather than the resilient group, by a factor of 0.97. However, age was not significant when psychosocial factors were added to the model. Alternatively, being female increased the relative risk of belonging to the predominantly depressed group, rather than the resilient group, by a factor of 2.78. Adding in the geographic variables in step two did not change this pattern, however the relative risk for sex did increase to 2.94. Adding the final step (psychosocial factors) shifted the pattern of significance. Identifying as female (4.55), COVID-19 rate (1.00), experiencing disruption as a result of COVID-19 (1.45), food access (1.81), access to information (1.72), and previous trauma history (1.72) all increased the relative risk of belonging to the predominately depressed class as compared to the resilient class. Alternatively, feeling that you have close connections in the community decreased the relative risk of belonging to the predominantly depressed class by 0.53.

#### Predominantly anxious class

In the predominantly anxious group, an additional year of age decreased the relative risk of belonging to the predominantly anxious group rather than the resilient group, by a factor of 0.97. Being White increased the relative risk by a factor of 2.78. Adding in the geographic variables in step two resulted in a change in the pattern of significance such that age (0.97), race (2.30), income (1.10), and rate of COVID-19 (1.00) in participants geographical region were all significant. An increase in income level increased the relative risk of belonging to the predominantly anxious class (as compared to the resilient class) by 1.10. Similarly, an increase in the COVID-19 rate increased the relative risk for the predominantly anxious class by a factor of 1.00. In the third step, age (0.97) and reporting close connections with the community (0.75) decreased the relative risk of belonging to the predominately anxious class as compared to the resilient class. Alternatively, income levels (1.16), race (2.69), and the COVID-19 infection rate (1.00) all increased the relative risk of belonging to the predominately anxious class.

#### Comorbid class

Education level was only significant for the comorbid group, such that one level higher of education decreased the relative risk of belonging to the comorbid group, rather than the resilient group, by a factor of 0.66. Adding in the geographic variables in step two did not change the pattern of significance though the relative risk ratio for education decreased slightly to 0.64. Education was no longer significant in the third step when adding in psychosocial variables. Identifying as female (5.65) experiencing COVID disruption (1.36), and having a trauma history (2.17) all increased the relative risk of belonging to the comorbid latent class. Alternatively, access to financial resources (0.44) and reporting close connections in the community (0.53) decreased the relative risk of belonging to the comorbid latent class as compared to the resilient one. Table 4 presents coefficients from the regression models including only significant predictors.

**Table 4:** Multinomial logistic regression predicting class membership.

		Block 1: Demographics	Block 2: Geographic	Block 3: Psychosocial
		RRR (se)	RRR (se)	RRR (se)
Predominantly depressed	Age	0.97 (0.01)*	0.97 (0.01)*	0.98 (0.01)
	Marital	0.68 (0.24)	0.69 (0.25)	0.73 (0.30)
	Race	1.07 (0.44)	1.15 (0.48)	0.93 (0.45)
	Education level	0.87 (0.11)	0.85 (0.11)	0.96 (0.14)
	Female	2.78 (1.40)*	2.94 (1.50)*	4.55 (2.80)*
	Income	1.00 (0.05)	1.01 (0.05)	1.07 (0.07)
	Viral rate	-	1.00 (0.00)	1.00 (0.00)**
	Rural	-	1.00 (0.01)	1.00 (0.01)
	Interdependence	-	-	1.07 (0.09)
	Disruption	-	-	1.45 (0.14)***
	Stressors	-	-	0.89 (0.12)
	Trauma history	-	-	1.72 (0.35)**
	Financial resources	-	-	0.69 (0.17)
	Social support	-	-	0.53 (0.09)***
	Food insecurity	-	-	1.81 (0.51)*
	Information access	-	-	1.72 (0.44)*
Predominantly anxious	Age	0.97 (0.01)**	0.97 (0.01)**	0.97 (0.01)*
	Marital	0.65 (0.19)	0.62 (0.19)	0.63 (0.21)
	Race	2.30 (0.92)*	2.63 (1.08)*	2.69 (1.20)*
	Education level	0.93 (0.10)	0.89 (0.10)	0.92 (0.11)
	Female	1.76 (0.66)	1.88 (0.73)	2.19 (0.92)
	Income	1.07 (0.05)	1.10 (0.05)*	1.16 (0.06)**
	Viral rate	-	1.00 (0.00)**	1.00 (0.00)***
	Rural	-	1.01 (0.01)	1.01 (0.01)
	Interdependence	-	-	1.09 (0.07)
	Disruption	-	-	1.11 (0.08)
	Stressors	-	-	1.11 (0.12)
	Trauma history	-	-	1.24 (0.21)
	Financial resources	-	-	0.71 (0.16)
	Social support	-	-	0.75 (0.11)*
	Food insecurity	-	-	1.58 (0.42)
	Information access	-	-	1.10 (0.22)
Comorbid	Age	0.98 (0.02)	0.98 (0.02)	0.98 (0.02)
	Marital	1.02 (0.48)	1.02 (0.48)	1.12 (0.63)
	Race	1.02 (0.54)	1.05 (0.56)	0.67 (0.41)
	Education level	0.66 (0.09)**	0.64 (0.09)**	0.72 (0.12)
	Female	2.56 (1.60)	2.63 (1.66)	5.65 (4.44)*
	Income	0.094 (0.07)	0.95 (0.07)	1.05 (0.09)
	Viral rate	-	1.00 (0.00)	1.00 (0.00)
	Rural	-	1.00 (0.02)	1.00 (0.02)
	Interdependence	-	-	0.92 (0.10)
	Disruption	-	-	1.36 (0.17)*
	Stressors	-	-	0.99 (0.17)	
	Trauma history	-	-	2.14 (0.52)**
	Financial resources	-	-	0.44 (0.13)**
	Social support	-	-	0.53 (0.12)**
	Food insecurity	-	-	1.55 (0.50)
	Information access	-	-	1.90 (0.64)

* *p* < 0.05 ** *p* < 0.01 *** *p* < 0.001.

## Discussion

Psychosocial factors have also been found to enhance resilience, including adequate information, the ability to meet basic needs and social support (d’Errico et al., 2018; Killgore et al., 2020; Labrague and J. A. A., 2020). This study explored psychological and social determinants of adaptation, to identify contributors to resilience activation. The four-factor solution, consistent with other post disaster studies (Osofsky et al., 2017), revealed four subgroups of adaptation including depressed, anxious, comorbid, and resilient. The majority of participants were classified into more negative symptom groupings (i.e., depressed, anxious, or comorbid). While alarming, these rates are consistent with other studies (Killgore et al., 2020), showing lowered resilience as a result of the pandemic. Importantly, analysis do not suggest the absence of resilience, rather anxiety and depression were more predominant; yet this presents an opportunity to identify target areas or variables that can strengthen resilience and decrease negative symptoms for future disasters.

### Resilience activation

Given the uncertainties, life changes, and fear associated with the COVID-19 pandemic, psychological concerns were expected (Hansel et al., 2020). Participants reported COVID-19 resulted in a loss of their usual way of life, social isolation, and working from home. Further confirming expectations, the higher number of stressors resulted in increased anxious and depressive symptoms. However, these findings did not hold in the multivariate analyses, suggesting that the resilient group, experienced psychological resilience rather than less stressors. Consistent with the definition of resilience, exposure must be similar and the ones who are resilient are those who rebound or bounce back from adversity with lower mental health symptoms (Bonanno et al., 2007). Multivariate analysis revealed four main areas to focus efforts for resilience activation improvement - these areas include chronic stress, finances, loneliness and information access.

#### Chronic stress

Findings from this study highlight the importance perceived chronic stress had on psychological determinants. Specifically, higher perceived life disruption increased the risk of belonging to both the depressed and comorbid groups. While quantity of pandemic related stressors were not predictive, perceived impact on life and work, suggestive of chronic stress environments, were. Increased serious mental illness or severity of mental health problems are common following other types of disasters and in part are contingent on exposure and recovery environment (Norris et al., 2002; Kessler et al., 2008). Similarly, to chronic stress in the recovery environment, previous trauma history increased the risk of being classified in the depressed and comorbid groups rather than in the resilient group, highlighting the impact of cumulative trauma and chronic stressors on mental health, (Lahav, 2020; Ran et al., 2020). Consistent with past disasters, the 2020 biological disaster highlights those psychosocial factors, including previous trauma, continue to disadvantage certain groups toward poorer mental health. Mental health services should be available for individuals with chronic stress, prior to and after disasters.

#### Finance

Access to financial resources and higher education increased the likelihood for being in the resilient group as compared to the comorbid group. In the multivariate analyses, higher income (based on reported income) resulted in decreased risk of belonging to both the depressed and anxious groupings as compared to resilience. Access to financial resources and the ability to buy food before running out was also a factor in group membership, where food access resulted in an increased risk of belonging to the depressed category. As with other disasters, individuals and communities with fewer resources tend to fare worse, highlighting that ways in which disasters disproportionately impact low or under resourced communities, as well as the importance of financial security on resilience as a major framework for resilience activation (Abramson et al., 2015; Cross et al., 2018; Gibbs et al., 2018; Maynard et al., 2018). In addition to mental health services, financial support and other resources should be a priority in disaster response.

#### Loneliness

Social cohesion has been shown to be a protective factor following the SARS pandemic (Lau et al., 2005). Our study supports the importance of community closeness, where feelings of close connections in the community and social support increased the likelihood of membership in resilient group, compared to the depressed, anxious and comorbid groups. Loneliness and the disruption of social support has been noted as a likely precursor to poor mental health outcomes (Saltzman et al., 2020). Social support safety nets can also buffer the effects of decreased financial and food access, linking loneliness and financial insecurity (Brück et al., 2019). Unique to more recent disasters, is the wide access to technology that may help buffer mental health problems, especially loneliness (Saltzman et al., 2017), but current findings were conflicting on the importance of information access.

#### Information access

Access to information increased the relative risk of belonging to the depressed and comorbid classes. This finding was unexpected, as access to information has been shown to be a protective factor in mental health risk (Reynolds et al., 2007). It may also suggest people with more experiences and disruption are seeking information and the quantity or quality of information is insufficient (Hansel et al., 2020; Legido-Quigley et al., 2020). A study also found that information following emergencies and disasters was most effective when it was timely, from a reliable or known source, and communicated in the recipient’s native language (Ichihara et al., 2016). However, the COVID-19 Pandemic presented an unprecedented scenario given the evolving and protracted nature of the crisis straining the ability of information sources to provide the most current and accurate information. 2020 underscored the growing role of social media and informal media as sources of information; less regulated media sources and a protracted public health crisis, in combination, was a previously untested scenario perhaps explaining the paradoxical findings in our data. In addition, studies have found associations among increase media usage and health anxiety (Mertens et al., 2020) as well as thee ongoing criticism around communication and the pandemic eroding the trustworthiness of several sources of information (Tanne, 2022). Given the context and the results of this study, we suggest that more research is needed to fully appreciate the complex role of information in protracted crises and we highlight an urgent need to develop further supports to improve information access that may support resilience in those scenarios.

### Social determinants

Other social determinant factors, such as demographics and community profiles, play a role in resilience activation. Age was confirmed as a protective factor in the multivariate results, where increased age decreased the likelihood of belonging to the depressed and anxious classes, thus older participants were more resilient. Other recent studies found youth were more susceptible to poorer mental health during the pandemic (Wilson et al., 2020; Browning et al., 2021). Similarly, being female increased the risk of being in the depressed or comorbid groups, a finding which has repeatedly been supported by past and recent literature (Barzilay et al., 2020; Xiong et al., 2020; Zhou et al., 2020). Conversely, identifying as White decreased the results of membership in the depressed or anxious groups, supporting concerns about growing health disparities (Bui et al., 2021; Masters et al., 2021; Young et al., 2021). Unsurprisingly, community indicators (i.e., viral rate) were predictive of belonging to both depressed and anxious, as compared to the resilient group, suggesting geographic location as a social determinant of resilience. Importantly, demographics and social determinants will vary greatly—while not specific to gender, age, and community indicators, service and resource allocation should be mindful of these indicators for implementation.

## Limitations and future research

Findings of this study provide insight into risk and protective factors that support resilience; however, it is not without limitations. Caution should be given toward interpretation of information access, given the lack of specificity around the single item. In addition, unequal and relatively small class sizes raise limitations regarding the interpretation of the multinomial regression results. Of particular note is the concern regarding statistical power for the comorbid latent class (*n* = 30). Although we note this limitation, we retain the 4-factor solution because the group sizes reflect expectations regarding the distribution of symptomology in the population (i.e., comorbid mental health concerns would be observed in the fewest number of people) and the overall sample size (*n* = 334) is sufficient for multinomial regression. Future studies are needed to understand the longer term and often changing profiles of resilience. Other limitations include the exploratory design, cross-sectional data, reliance on self-report measures and lack of generalizability. Despite limitation, similar patterns of associations are likely and expected to hold—if not strengthen—with more representative samples. Future research should assess biological indicators of resilience and the function of competency and autonomy in the disaster recovery process (Diotaiuti et al., 2021c).

## Conclusion

Similar to the social determinants of health (World Health Organization, 2022), resilience activation frameworks (Abramson et al., 2015), and the five psychosocial elements of trauma intervention (Hobfoll et al., 2007), this study identified factors that contribute to overall wellbeing despite chronic stressors. Social determinants of adaptation, found in this study population, include loneliness, finances, and information access. Economic security is the most challenging, but access to education, training, financial literacy and equitable resource distribution, are likely to increase resilience. Reporting closeness with community consistently decreased the relative risk of belonging to less favorable adaptation profiles suggesting that community cohesion and social support play an important role in building resilience. Information access, both quality and quantity, is a major factor in resilience. Knowing the importance of information access can be used in recovery efforts to communicate psychoeducation inclusive of resilience. The findings from this study support the need for both psychological and social adaption supports, inclusive of mental health treatment, to strengthen resilience activation.

## Data availability statement

The raw data supporting the conclusions of this article will be made available by the authors, without undue reservation.

## Ethics statement

The studies involving humans were approved by Tulane University Human Research Protection Office. The studies were conducted in accordance with the local legislation and institutional requirements. The ethics committee/institutional review board waived the requirement of written informed consent for participation from the participants or the participants’ legal guardians/next of kin because consent was the only way to track participants and thus to ensure anonymity the IRB granted a written consent waiver.

## Author contributions

LS and TH collaboratively worked on the entire manuscript, conception, data collection, data analysis, and writing. All authors contributed to the article and approved the submitted version.
